# Pain-guided activity modification during treatment for patellar tendinopathy: a feasibility and pilot randomized clinical trial

**DOI:** 10.1186/s40814-021-00792-5

**Published:** 2021-02-25

**Authors:** Andrew L. Sprague, Christian Couppé, Ryan T. Pohlig, Lynn Snyder-Mackler, Karin Grävare Silbernagel

**Affiliations:** 1 Department of Physical Therapy, University of Delaware, Newark, DE USA; 2 Department of Biomechanics and Movement Science, University of Delaware, Newark, DE USA; 3 Department of Physical Therapy, University of Pittsburgh, Pittsburgh, PA USA; 4 Department of Orthopaedic Surgery M, Faculty of Health and Medical Sciences, Institute of Sports Medicine Copenhagen, Bispebjerg Hospital and Center for Healthy Aging, University of Copenhagen, Copenhagen, Denmark; 5 Department of Physical and Occupational Therapy, Bispebjerg Hospital, Copenhagen, Denmark; 6 IOC Research Center Copenhagen Center for Injury Prevention and Protection of Athlete Health, Bispebjerg Hospital, Copenhagen, Denmark; 7 Biostatistic Core Facility, College of Health Sciences, University of Delaware, Newark, DE USA; 8 Department of Biomedical Engineering, University of Delaware, Newark, DE USA

**Keywords:** Patellar tendinopathy, Jumper’s Knee, patellar tendinitis, Activity modification, Elastography, Exercise therapy, Tendon loading

## Abstract

**Background:**

Activity modification is a key component of patellar tendinopathy treatment but there is a lack of evidence guiding activity modification prescription. Use of activity modification in treatment studies has varied widely and the impact of those recommendations has not been directly investigated or compared. The purpose of this study was to assess (1) the feasibility of using pain-guided activity modification during treatment for patellar tendinopathy and (2) if our outcome measures are responsive to changes in tendon health over the course of treatment.

**Methods:**

This was an unblinded, randomized two-arm pilot and feasibility study randomized clinical trial with parallel assignment, conducted in Newark, DE. Individuals between the ages of 16 and 40 years old with patellar tendinopathy were included. Participants were randomly assigned to a *pain*-*guided activity* (*PGA*) or *pain*-*free activity* (PFA) group using a spreadsheet-based randomization scheme*.* All participants received standardized treatment using a modified version of the heavy-slow resistance protocol 3×/week for 12 weeks. For the first 6 weeks, the PGA group used the Pain-Monitoring Model to guide activity outside of treatment and the PFA group was restricted from running, jumping, or activities that provoked their patellar tendon pain. Feasibility outcomes included recruitment, enrollment, randomization, compliance, and retention percentages. Clinical evaluations were conducted at baseline, 6, and 12 weeks to assess symptom severity, psychological factors, tendon morphology and mechanical properties, lower extremity function, and quadriceps muscle performance.

**Results:**

In a ~ 13-month period, 108 individuals were screened, 47/108 (43.5%) were eligible for participation, and 15/47 (32.0%) of those were enrolled (9 PGA, 6 PFA). The recruitment rate was 1.15 participants/month. The mean ± SD compliance with treatment was PGA: 86.1 ± 13.0% and PFA: 67.1 ± 30.7%. There was one missed evaluation session and two adverse events, which were not due to study interventions. Changes exceeding the smallest detectable change were observed for at least one outcome in each domain of tendon health.

**Conclusions:**

Use of pain-guided activity modification during exercise therapy for patellar tendinopathy was found to be feasible, and the proposed outcome measures appropriate. Computer-based allocation concealment, blinding of evaluators, and greater recruitment of high-level athletes should be implemented in future trials.

**Trial registration:**

ClinicalTrials.gov identifier: NCT03694730. Registered 3^rd^ of October, 2018.

**Supplementary Information:**

The online version contains supplementary material available at 10.1186/s40814-021-00792-5.

## Key messages regarding feasibility

- The feasibility of pain-guided activity modifications recommendations for athletes with patellar tendinopathy is unclear, as pain-guided activity modification uses an individualized approach. However, these patients typically participate in team sports where they may not have control of their training schedule. Additionally, this study included novel measures of tendon health and it is unknown whether these measures would be responsive to changes over 12 weeks of treatment.
- The use of pain-guided activity modification for athletes with patellar tendinopathy appears feasible since self-reported compliance was high. Furthermore, the proposed outcome measures were responsive to changes in tendon health over the course of treatment.
- These findings suggest that a full clinical trial should be pursued, although alterations are needed to allocation concealment, blinding of evaluators, and recruitment to limit bias.

## Background

Patellar tendinopathy is an overuse injury to the patellar tendon, usually resulting from excessive overload to the tendon with inadequate time for recovery [1]. Therefore, activity modification is considered a key component of patellar tendinopathy treatment [2]. Activity modification is usually paired with exercise therapy, consisting of tendon loading exercises, which is the treatment that currently has the highest level of evidence supporting its efficacy [3, 4]. Clinicians typically modify the intensity, duration, and/or type of activities performed outside of treatment to ensure that the tendon can sufficiently recover between bouts of tendon loading exercises [1].

Although activity modification is recognized as a key component of patellar tendinopathy treatment, implementation of activity modification in treatment studies varies widely. Recommendations for altering recreational physical activity have ranged from complete cessation of sports participation [5–7] to continued activity at the pre-injury level [8–10]. Other studies have used a more individualized approach where pain levels are used to guide activity modification [11, 12]. Each of these approaches have yielded positive results; however, the impact of activity modification on clinical outcomes have not been directly investigated nor compared. Therefore, it is unclear whether activity modification recommendations were beneficial or detrimental to clinical outcomes.

The optimal amount of activity modification likely lies somewhere between activity cessation and full participation since either end of the spectrum may have negative consequences. Absence from sport due to injury has been associated with increased anxiety, depression, and reduced self-esteem [13]. Additionally, activity cessation may reduce the tendon’s tolerance to sport-specific loads and decrease physical fitness. As a result, these athletes may be at increased risk for re-injury or the development of new injuries. On the other hand, full participation may reduce or nullify the benefits of exercise therapy, as patients lack the necessary recovery time for tendon remodeling [1, 8]. Therefore, it is of interest to identify a middle ground between activity cessation and full participation, which limits negative psychological consequences of injury, maintains physical readiness for sport, and maximizes recovery.

Pain-guided activity modification using the Pain-Monitoring Model may be a suitable middle ground for activity modification. Originally described by Thomeé et al. for use in patellofemoral pain, the Pain-Monitoring Model provides guidelines for acceptable pain levels during activity [14]. Briefly, pain levels should not exceed a 5/10 on the numeric pain rating scale (NPRS) during or immediately after activity. Additionally, pain ratings should return to pre-activity levels by the following morning. In a prior randomized clinical trial (RCT), the Pain-Monitoring Model was adapted for use in Achilles tendinopathy [15]. In this study, continued activity using the Pain-Monitoring Model had no detrimental effects on clinical outcomes when compared to a group with pain-free activity modification. This approach to activity modification has been previously utilized for patellar tendinopathy; however, the impact on clinical outcomes has not been investigated [11, 12].

Ultimately, our research group aims to compare the impact of continued activity using the Pain-Monitoring Model on clinical outcomes in patellar tendinopathy with other approaches to activity modification. Such a study has the potential to improve activity modification recommendations and balance the need for recovery with the physical and emotional health of athletes with patellar tendinopathy. However, prior to initiating a large RCT, it is critical to establish the feasibility of the study design [16]. This is especially important in patellar tendinopathy, since this injury is most common in team sports and using individualized activity recommendations may pose unforeseen challenges. Additionally, it is important to assess whether the proposed outcome measures are able to capture changes in response to the intervention. This ensures that the participants are not subjected to undue burden and that resources are allocated to worthwhile measures. Therefore, the purpose of this study was two-fold:

1. To assess the feasibility of a randomized clinical trial utilizing pain-guided activity modification while undergoing standardized treatment for patellar tendinopathy. As part of this aim, we will examine:
  - Access to potential participants
  - Percentage of potential participants meeting inclusion criteria and percentage of eligible participants willing to be randomized
  - Monthly recruitment
  - Retention
  - Compliance with treatment, activity modification, and training diaries
2. To evaluate if the chosen primary and secondary outcome measures are responsive to changes in tendon health over the course of treatment.

## Methods

### Study design

This study was a pilot and feasibility randomized clinical trial with participants randomized into two arms with parallel assignment and balanced allocation. The study was approved by the University of Delaware Institutional Review Board, prospectively registered (ClinicalTrials.gov ID: NCT03694730, Registered: October 3^rd^, 2018), and conducted according to CONSORT guidelines [17, 18]. To be included, participants had to have a clinical diagnosis of patellar tendinopathy and be between the ages of 16 and 40 years old. The diagnostic criteria for patellar tendinopathy was (1) pain and stiffness isolated to the patellar tendon and (2) load-dependent symptoms, which increased as the demands on the patellar tendon increased [2, 19]. Potential participants were excluded if they had an injury, other than patellar tendinopathy, that limited their ability to participate in treatment and/or testing. Additionally, individuals were excluded if they had knee surgery or an injection, tenotomy, or shockwave to the patellar tendon within the last 6 months. All participants received written and oral information about the study prior to participation and provided written informed consent.

**Fig. 1:**
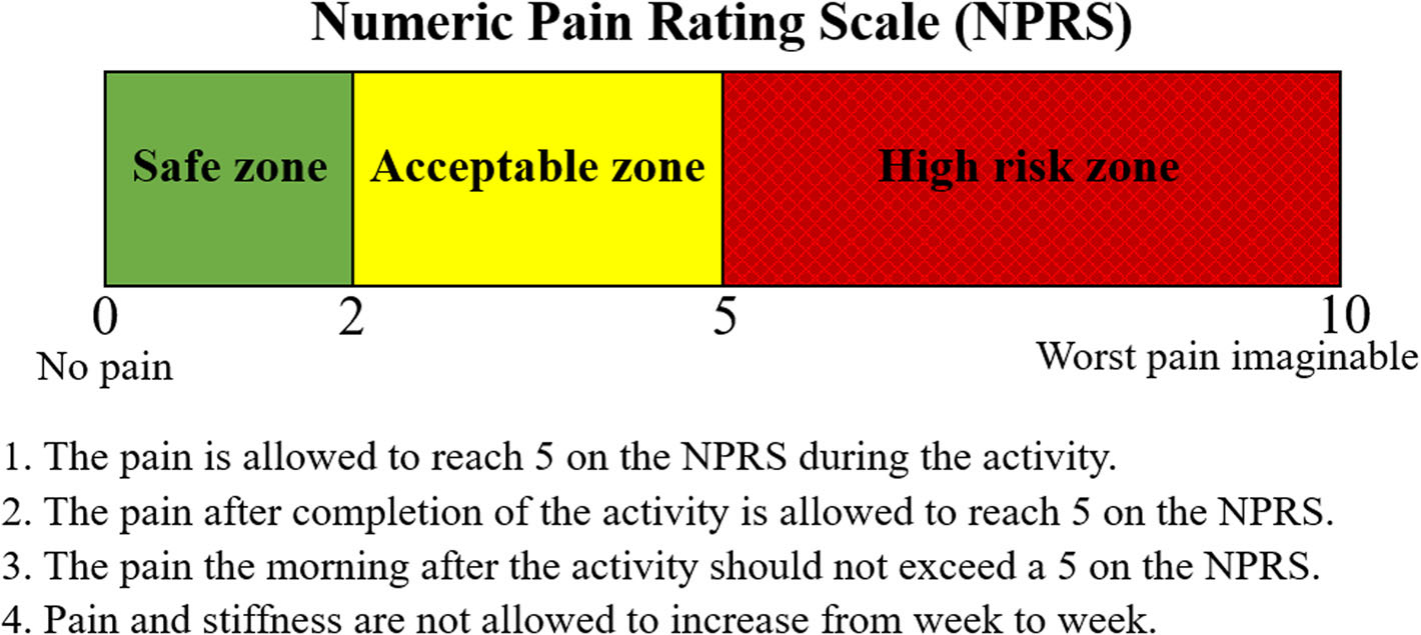
Pain-monitoring model

Participants were randomly assigned into one of two treatment groups: (1) a *Pain*-*Guided Activity* (PGA) group and (2) a *Pain*-*Free Activity* (PFA) group. All participants completed patellar tendon loading exercises three times a week for 12 weeks using a standardized treatment program [11, 12]. During the first 6 weeks of treatment, participants were asked to limit their physical activity outside of treatment, in accordance with their treatment strategy. Participants in the PGA group were allowed to continue their usual recreational activities, using the pain-monitoring model to guide activity intensity (Fig. 1) [15]. The PFA group were not allowed to perform running, jumping, or activities that provoke their patellar tendon pain outside of treatment. However, they could perform activities that were not pain-provoking, such as swimming or the elliptical. Additionally, participants completed evaluations at baseline, 6, and 12 weeks to assess symptom severity, quality of life, psychological factors, tendon morphology and mechanical properties, lower extremity function, and quadriceps muscle performance. All supervised treatments and evaluation sessions were completed at the University of Delaware Health Sciences Complex in Newark, DE. Enrollment, randomization, evaluations, and treatment were all completed by the same physical therapist precluding blinding.

### Recruitment and randomization

Participants were recruited from the University of Delaware community and the surrounding region between January 2019 and February 2020. A recruitment strategy was developed that incorporated multiple media platforms and recruitment locations to ensure a representative sample of individuals with patellar tendinopathy (Supplementary Figure S.1). Interested individuals were directed to an online screening questionnaire for preliminary eligibility assessment. Following completion of this questionnaire, interested individuals were contacted by a member of the research team to provide additional information about the study, clarify responses on the eligibility questionnaire, and schedule their in-person screening and informed consent. Access to potential participants, results of eligibility screening, and willingness to be randomized were recorded using Research Electronic Data Capture (REDCap) (Vanderbilt University, Nashville, TN) [20]. Additionally, recruitment sources and reasons for ineligibility or declined participation were recorded.

The randomization scheme was generated by a biostatistician and stratified for sex without blocking. The randomization scheme was stored as a spreadsheet on a password protected computer. Participants were randomized after providing informed consent and completing the baseline assessment. The participants were notified of their group assignment at the first treatment session by the treating physical therapist.

### Treatment protocol

**Fig. 2:**
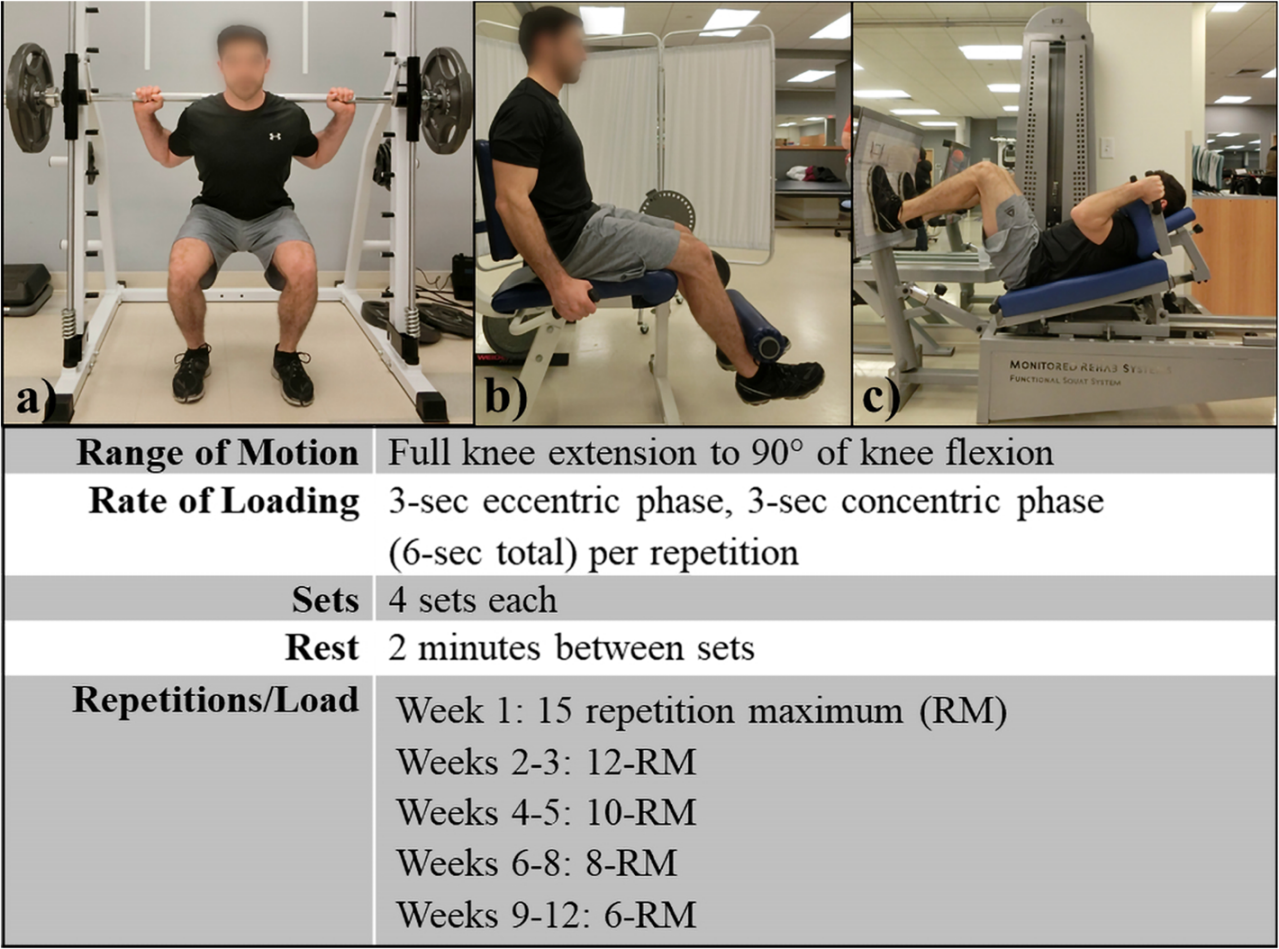
Heavy-slow resistance training parameters for the **a** squat, **b** knee extension, and **c** leg press

All participants completed a modified version of the Heavy-Slow Resistance protocol three times a week for 12 weeks [11, 12]. The original protocol consists of three exercises, the squat, leg press, and hack squat. In the modified version, the hack squat was replaced with the knee extension due to equipment availability. Participants complete four sets of each exercise per session using a 6-s count per repetition (3 s eccentric, 3 s concentric phase). An auditory metronome was used to pace each repetition. Over the course of the protocol, the load is progressively increased, and repetitions are decreased (Fig. 2). Resistance levels for each phase of the treatment protocol are dosed based on the participants’ 5-repetition max (5RM) for each exercise, which were performed at the initial treatment and approximately every 2 weeks after. All supervised treatments were completed by the same physical therapist and participants were required to attend at least one supervised session every 2 weeks to complete 5RM testing. Since patellar tendinopathy typically occurs in athletic individuals who are familiar with strength training, participants were given the option to complete other treatments at their personal gym or team facility. At 6 and 12 weeks, participants were asked to rate their level of satisfaction on a 10-point Likert scale (0 = not satisfied, 10 = very satisfied).

### Compliance, retention, and safety

Compliance with exercises and activity modifications were tracked using paper training diaries. For each day, participants were asked to record the treatment exercises completed, whether they performed running and/or jumping activity, any other physical activity performed, and their pain levels upon waking. Furthermore, if participants performed physical activity other than treatment exercises, they were asked to rate their pain levels prior to, during, and after the activity. The training diaries were collected weekly and reviewed by the research team. For each week, the number of times treatment exercises were completed, days the training diary was completed, days of running or jumping activity, and days in compliance with activity restrictions for weeks 1 through 6 were recorded. Participants were considered compliant with treatment if they performed at least two of the three prescribed exercise sessions per week. Additionally, the number of missed follow-up evaluations, drop-outs, and adverse events were recorded.

### Clinical outcomes

The primary clinical outcome for this trial was symptom severity, assessed by the Victorian Institute of Sports Assessment–Patellar Tendon (VISA-P) questionnaire. All other clinical outcomes served as secondary outcome measures. If participants had bilateral symptoms, they were asked to identify their most symptomatic limb in their baseline questionnaires and the most symptomatic limb was examined.

#### Symptom severity

Symptom severity was assessed using the VISA-P and palpation. The VISA-P is an 8-item questionnaire designed to assess patellar tendinopathy symptom severity and the impact on physical function [21]. Scores range from 0 to 100 with lower scores indicating greater disability. This instrument has a minimally clinically important difference (MCID) of 13 points [22]. Palpation was performed along the length of the patellar tendon and at the patellar and tibial insertions. Participants were asked to rate their pain during palpation on the NPRS. The NPRS has an MCID of 1.7 points [23].

#### Quality of life

Knee-related quality of life was measured using the Knee injury and Osteoarthritis Outcome Score–Quality of Life subscale (KOOS-QOL) [24]. Scores on this subscale range from 0 to 100%, with higher scores indicating better knee-related quality of life.

#### Psychological factors

Participant’s fear of movement and re-injury, or kinesiophobia, were captured using the Tampa Scale of Kinesiophobia (TSK-17) [25, 26]. Higher levels of kinesiophobia have been associated with worse recovery of lower extremity function in Achilles tendinopathy [27]. Additionally, in pilot studies, we found that the majority of patients with patellar tendinopathy have clinically meaningful levels of kinesiophobia [28]. Scores range from 17 to 68 points, with higher scores indicating a greater fear of movement and re-injury.

Participant’s magnified negative orientation toward pain, or pain catastrophizing, was recorded using the Pain Catastrophizing Scale (PCS) [29]. Scores range from 0 to 52 points, with higher scores indicating a greater degree of pain catastrophizing.

The presence and severity of negative emotional states was measured using the Depression, Anxiety, and Stress Scale (DASS-21) [30]. The DASS-21 has previously been used to evaluate the mental health of athletes at a variety of competition levels [31, 32]. The DASS-21 consists of 21 questions that can be divided into three subscales, (1) Depression, (2) Anxiety, and (3) Stress. Scores range from 0 to 63 points, with higher scores indicating a greater degree of negative emotional states.

#### Tendon morphology

B-mode ultrasound imaging was performed at the patellar tendon using a LOGIC *e* Ultrasound (GE Healthcare, Chicago, IL) system with a wide-band linear array probe (5.0–13.0 MHz) at 10 MHz and 2.5 cm depth to assess tendon morphology. Participants were positioned in supine with the knee flexed to 30° and supported by a bolster [33]. Three extended field of view long-axis images were completed from the tibial tuberosity to the inferior pole of the patellar to obtain maximal tendon thickness. Additionally, three short-axis images were taken at 1 cm distal to the inferior pole of the patella to obtain cross-sectional area (CSA). A custom MatLab code was used to identify the maximal tendon thickness and Osirix MD imaging software (Pixmeo, Geneva, Switzerland) was used to measure CSA. The average of three images was used for analysis.

#### Tendon mechanical properties

Continuous shear wave elastography (cSWE) was used to evaluate patellar tendon mechanical properties with a Sonix MDP Q+ (Ultrasonix, Vancouver, BC, Canada) ultrasound scanner with a L14-5/38 probe [34, 35]. For this technique, participants were seated on an adjustable plinth with their legs stabilized at 90° of hip and knee flexion. The inferior pole of the patella and the tibial tuberosity were identified, and a mark was placed 1 cm distal to the inferior pole of the patella, along the imaginary line connecting the two bony landmarks. The ultrasound probe was centered over this mark, in line with the long axis of the tendon. A Minishaker Type 4810 (Bruel and Kjaer, Norcross, GA, USA) was placed on the quadriceps tendon and used to produce shear waves at 11 different frequencies (322, 339, 358, 379, 402, 429, 460, 495, 536, 585, and 643 Hz). As each frequency propagated through the patellar tendon, the ultrasound probe captured raw radiofrequency data at 6438 frames/s. A custom MATLAB code was used for post-processing to provide estimates of static shear modulus and viscosity, as described by Cortes et al and Corrigan et al. [34, 35]. Three trials were performed and the average of three trials was used for analysis.

#### Lower extremity function

The single-leg counter-movement jump (CMJ) and single-leg drop CMJ were used to evaluate lower extremity function [36]. These tests have high reliability and have previously been used to assess function in lower extremity tendinopathies [15, 36, 37]. For the CMJ, participants began by standing on a single leg on flat ground with their hands behind their back. They were instructed to jump as high as they can, landing on the same leg with which they took off from the ground [36]. The drop CMJ was performed similarly except that participants assumed the starting position on a 20 cm high box. They were instructed to “drop” off of the box and then jump as high as they can once they contacted the ground [36]. For both tests, an infrared light mat (MuscleLab®, Ergotest Innovations, Stathelle, Norway) was used to record flight time, which was then used to estimate jump height. The average of three trials was used for analysis.

#### Quadriceps muscle performance

A knee extension maximal voluntary isometric contraction (MVIC) with the burst-superimposition method was used to evaluate knee extension strength and quadriceps muscle activation [38]. This technique has demonstrated reliability and has been utilized in a variety of chronic knee injuries [38–45]. Participants were seated on a KinCom dynamometer (Model 50 H, Isokinetic International, Chattanooga, TN, USA) at 90° of hip flexion and 60° of knee flexion for the tested limb. Self-adhesive electrodes were placed over the distal vastus medialis and proximal vastus lateralis muscle bellies. After familiarization with procedures and a standardized warm-up, participants were instructed to perform a 5-s MVIC. During the MVIC, a supramaximal, 10-pulse (600 μs, 130 V, 100 pulses per second) train of electrical stimulation was applied to the muscle using an electrical stimulator (Grass Technologies, Champaign, IL). Verbal encouragement was provided throughout each trial. If the participant was unable to activate quadriceps fully or they did not reach and maintain their peak MVIC prior to delivery of the burst, testing was repeated up to 4 times, with 3-min rest between trials. The MVIC force and force attributable to the electrical stimulation was recorded. The best trial, based on force production and visual inspection of the force production graph, was selected to calculate quadriceps central activation ratios (CAR = [MVIC force/burst augmented force] × 100%). The CAR is a measure of quadriceps inhibition, where lower values indicate a greater degree of quadriceps inhibition.

### Alterations to study protocol after initiation

There were several changes to the study protocol after initiation due to staffing and equipment restraints, as well as the COVID-19 pandemic. First, it was our intention to use a blinded assessor for the baseline and follow-up assessments. Due to limited staff, all assessments and treatment were provided by the same physical therapist, which precluded blinding. Second, we intended to collect the mechanical pain threshold using a pain-pressure algometer as a secondary outcome measure. Due to equipment issues, this outcome was only collected for a small portion of the assessments. Therefore, we have chosen not to report these values. Third, in-person human subjects research was halted on March 17^th^, 2020 by the Institutional Research office at the University of Delaware due to the COVID-19 pandemic. At that time, there were seven active participants in the study. One had completed treatment and was scheduled for their 12-week follow-up; the remaining participants had completed their 6-week evaluations and were in the final phase of the treatment protocol. For those participants still in treatment, a modified version of the treatment protocol was created utilizing resistance bands, so participants could continue treatment without access to fitness facilities. Bands of varying resistance (Rogue Monster Bands, Rogue Fitness, Columbus, OH) were mailed to the participants to ensure that they could replicate the resistance of isotonic exercises as closely as possible. If possible, 12-week follow-up evaluations were completed remotely with the participants completing questionnaires online. Therefore, follow-up measures of tendon morphology and mechanical properties, lower extremity function, and quadriceps muscle performance were not collected for these participants at 12 weeks. Additionally, we intended to complete 6- and 12-month follow-ups for all participants. However, with ongoing restrictions due to the pandemic, this study was not approved to resume for later follow-ups since the participants were no longer in treatment and there was minimal direct benefit to participants. Therefore, 6- and 12-month follow-ups were not completed.

### Statistical analysis

Statistical analysis was performed using R version 3.6.3 and IBM SPSS version 26 (Chicago, IL) statistics software [46, 47]. The target sample size was determined a priori based on a minimally clinically important improvement of 13 points in the VISA-P from baseline to 12 weeks using values obtained from a prior study [11, 22]. It was determined that 10 participants would be required per group with 80% power and alpha set at 0.05. To account for drop-out, the target recruitment was set at 15 participants per group (30 total).

Descriptive statistics were calculated for demographic information at baseline and outcome measures at each timepoint for both groups. Additionally, descriptive statistics were calculated for results of recruitment, randomization, compliance, retention, and safety. A priori criteria were not established to determine if a full clinical trial should be conducted.

A 2 × 3 generalized linear mixed model (GLMM) was used to test the change over time for both groups for the primary outcomes: symptom severity, psychological factors, tendon morphology and mechanical properties, lower extremity function, and quadriceps muscle performance using the intention to treat principle [48–51]. Group (PGA or PFA) and time point (baseline, 6, and 12 weeks) were included as fixed effects. A compound symmetric covariance matrix was used to model the correlation among residuals. GLMM models can garner accurate estimates in the presence of missing data without excluding entire cases. Allowing for anyone with an observation at a time point to be included, assuming that data is at least missing at random [51].

To test the assumption of normality and to look for outliers, residuals were tested using Shapiro-Wilk tests, and screened for outliers. If time was significant, all pairwise comparisons were tested post-hoc. Mean differences among timepoints were compared to the smallest detectable change (SDC) and minimally clinically important difference (MCID) to assess the magnitude of effect for all outcomes (Supplementary Table S.1). Alpha was set at 0.05 for all tests.

## Results

### Recruitment

**Fig. 3:**
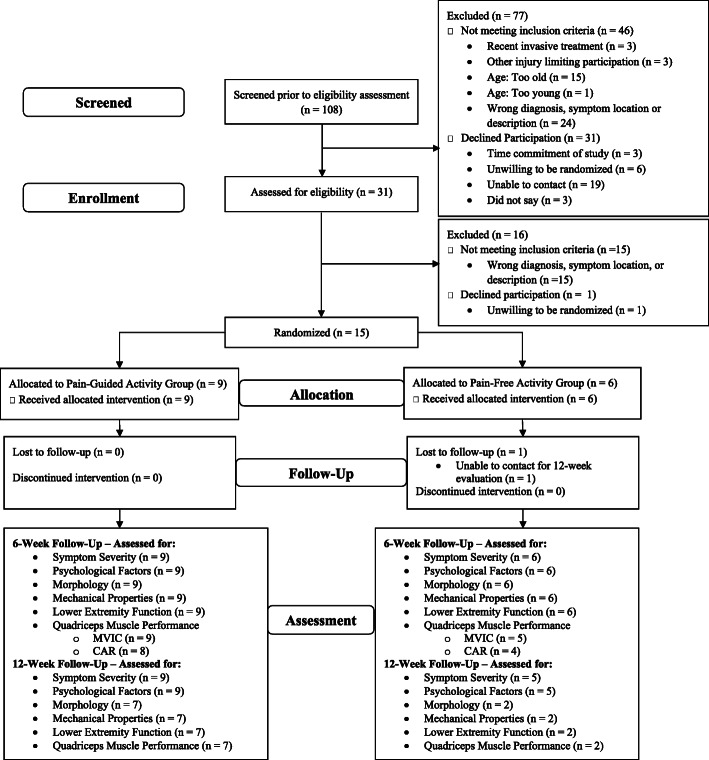
CONSORT flowchart for recruitment and randomization

Recruitment ran from January 15^th^, 2019 to February 1^st^, 2020, and follow-ups were completed on April 21^st^, 2020. The recruitment window was established a priori with a planned ending date of February 1^st^, 2020. At total of 108 individuals completed the initial screening online or by phone (Fig. 3). Of these individuals, 62 were deemed potentially eligible but only 31 agreed to in-person screening (Fig. 3). Following in-person screening, 16 individuals were deemed not eligible and one declined participation (Fig. 3). Fifteen participants were randomized (PGA: 9; PFA: 6). In total, 43.5% (47/108) of interested individuals were eligible for participation and 32.0% (15/47) of those that were eligible were willing to be randomized (Fig. 3). Monthly recruitment was 1.15 participants per month.

### Participants

Participant demographics and training history are summarized in Table 1. Participant demographics were similar between groups. However, the PGA group had shorter symptom duration and higher VISA-P scores than the PFA group. Additionally, the PGA group performed more strength training per week than the PFA group. There were two participants in the PGA group and three in the PFA group with bilateral symptoms. Fourteen of the fifteen participants competed in sports at the recreational level and the 15^th^ at the collegiate level.

**Table 1:** Baseline demographics and training history for pooled sample and group assignments

	Pooled Sample Mean (SD) Median (IQR) (*n* = 15, 5 F)	Pain-guided Mean (SD) Median (IQR) (*n* = 9, 3 F)	Pain-free Mean (SD) Median (IQR) (*n* = 6, 2 F)
Age (years)	26.6 (3.9) 27.0 (7.0)	26.9 (3.5) 28.0 (4.0)	26.3 (4.8) 25.0 (7.8)
Height (cm)	175.9 (9.8) 172.7 (13.0)	176.9 (11.0) 174.6 (4.0)	174.4 (8.4) 172.4 (7.3)
Weight (kg)	79.4 (16.9) 80.1 (19.6)	79.9 (12.7) 82.2 (5.5)	78.6 (23.3) 68.2 (20.4)
BMI (kg/m^2^)	25.5 (4.3) 24.7 (4.5)	25.4 (3.0) 25.9 (4.1)	25.6 (6.1) 23.5 (2.4)
Symptom duration (months)	32.2 (42.1) 15.8 (36.4)	21.2 (27.4) 7.5 (28.4)	48.8 (56.7) 28.4 (69.3)
VISA-P (points)	58.9 (17.9) 60.0 (19.0)	65.7 (15.8) 69.0 (20.0)	48.8 (17.1) 51.5 (18.3)
Weekly Hours Training/Playing Primary Sport (h/wk)	6.1 (5.2) 4.0 (5.0)	5.7 (5.9) 3.0 (5.0)	6.8 (4.5) 6.0 (4.3)
Weekly hours training/playing other sports (h/wk)	1.8 (1.9) 2.0 (1.0)	2.0 (2.4) 2.0 (1.0)	1.5 (1.0) 1.5 (1.0)
Weekly hours of jump training (h/wk)	1.0 (1.0) 1.0 (1.0)	1.3 (1.2) 1.0 (0.5)	0.5 (0.6) 0.5 (1.0)
Weekly hours of strength training (h/wk)	4.6 (4.1) 4.0 (3.5)	5.6 (4.7) 4.0 (4.0)	3.0 (2.4) 3.0 (3.5)

*BMI* Body mass index

### Compliance, retention, and safety

Participants’ compliance with treatment, training diaries, and activity restrictions are summarized in Table 2. There was one participant lost to follow-up from the PFA group who did not complete their 12-week evaluation questionnaires after in-person evaluations were halted due to COVID-19. Additionally, this participant had completed the treatment portion of the study but had not provided their training diaries. There were two adverse events during the study, both in the PFA group. In both instances, the participants experienced a new onset of lateral knee pain while performing activities unrelated to study participation, at their home or their gym. One participant slipped in the shower and the other participant hyperextended their knee while using the elliptical. However, in both cases, these new symptoms were aggravated by tendon loading exercises and required modification to knee angles and/or prescribed loads to limit discomfort. For both participants, symptoms resolved without additional treatment.

**Table 2:** Participants compliance with treatment, training diaries, activity modification, and satisfaction with treatment

	Pain-guided Mean (SD) Median (IQR)	Pain-free Mean (SD) Median (IQR)	Pain-free—complete cases^a^ Mean (SD) Median (IQR)
Proportion of prescribed treatment sessions completed (%)	86.1 (13.0) 83.3 (22.2)	67.1 (30.7) 77.8 (20.1)	78.9 (11.7) 80.6 (11.1)
Proportion of weeks compliant with treatment (%)	92.3 (9.4) 91.7 (8.3)	76.4 (37.8) 91.7 (6.3)	91.7 (5.9) 91.7 (0.0)
Proportion of days training diary was completed (%)	98.4 (2.7) 100.0 (2.4)	81.3 (40.0) 98.8 (7.7)	97.6 (4.1) 100.0 (2.4)
Proportion of days compliant with activity restrictions (%)	97.3 (3.9) 100.0 (5.4)	80.2 (39.8) 98.8 (13.1)	96.2 (7.3) 100.0 (2.4)
Days of running or jumping per week (days/wk) ^b^	1.9 (1.4) 1.5 (2.0)	0.0 (0.2) 0.0 (0.0)	0.0 (0.2) 0.0 (0.0)
Satisfaction with treatment at 6 weeks	9.0 (1.1) 9.0 (2.0)	7.3 (2.3) 7.0 (4.3)	
Satisfaction with treatment at 12 weeks	9.3 (1.1) 10.0 (1.0)	8.4 (2.1) 9.0 (2.0)	

^a^Participant that was lost to follow-up following COVID-19 was dropped for the complete cases analysis

^b^First 6 weeks used since restrictions on running and jumping were only applied for the first 6 weeks of treatment

### Satisfaction with treatment

Satisfaction with treatment at 6-week and 12-week follow-ups is summarized in Table 2.

### Clinical outcomes

The estimated marginal means, standard error, and pairwise comparisons of timepoints for the pooled sample are displayed in Table 3. The tests of model effects and results prior to outlier removal are displayed in the Supplementary Tables S.2, S.3, S.4.

**Table 3:** Estimated marginal means, standard error, and pairwise comparisons of timepoints for the pooled sample

Category	Outcome	Baseline		6 weeks		12 weeks^a^		*p* values		
		M	SE	M	SE	M	SE	Baseline to 6 weeks	Baseline to 12 weeks	6 weeks to 12 weeks
Symptoms	VISA-P (points)	59.6	3.8	68.8	3.8	75.6	4.0	**0**.**043**	**0**.**001**	0.136
	Palpatory pain (NPRS)	3.8	0.5	2.8	0.6	2.3	0.6	**0**.**010**	**0**.**006**	0.347
Quality of life	KOOS-QOL (%)	42.4	3.7	58.9	3.7	68.0	3.9	**< 0**.**001**	**< 0**.**001**	**0**.**029**
Psychological factors	TSK (points)	36.4	1.1	35.8	1.1	34.2	1.1	0.448	**0.015**	0.075
	DASS-21 (points)	3.2	1.0	3.4	1.1	5.6	1.1	0.847	0.040	0.061
	PCS (points) ^b^	2.0	6.0	1.0	2.0	1.0	3.0	–	–	–
Morphology	Thickness (mm)	6.9	0.5	6.8	0.5	6.6	0.6	0.427	0.329	0.678
	CSA (mm^2^)	111.9	11.0	118.0	10.6	118.0	12.0	0.355	0.443	1.000
Mechanical properties	Shear Modulus (kPa)	70.6	6.3	77.1	6.2	79.8	9.2	0.427	0.391	0.796
	Viscosity (Pa*sec)	31.6	2.4	29.7	2.3	33.0	3.1	0.396	0.628	0.268
Lower extremity function	CMJ height (cm)	12.1	1.2	13.3	1.2	13.7	1.3	**0**.**033**	**0**.**045**	0.620
	Drop CMJ height (cm)	13.4	1.2	13.9	1.3	13.4	1.4	0.423	0.953	0.588
Quadriceps muscle performance	MVIC (N)	860.9	56.7	995.9	60.0	1053.4	85.6	0.138	0.082	0.601
	CAR (%)	80.6	3.3	84.1	3.8	90.7	4.9	0.440	0.076	0.250

*CSA* Cross-sectional area, *CMJ* Counter-movement jump, *MVIC* Maximal voluntary isometric contraction, *CAR* Central activation ratio, *NPRS* Numeric pain rating scale

Bold indicates statistically significant differences between timepoints

^a^*n* = 14 for patient reported outcome measures at 12 weeks (VISA-P, TSK, PCS, DASS-21) due to participant drop-out. *n* = 8 for all other measures at 12 weeks due to inability to complete in-person follow-ups because of COVID-19

^b^PCS did not meet assumption of linearity so model significance was not tested and values reported are median and interquartile range, respectively

#### Symptom severity

**Fig. 4:**
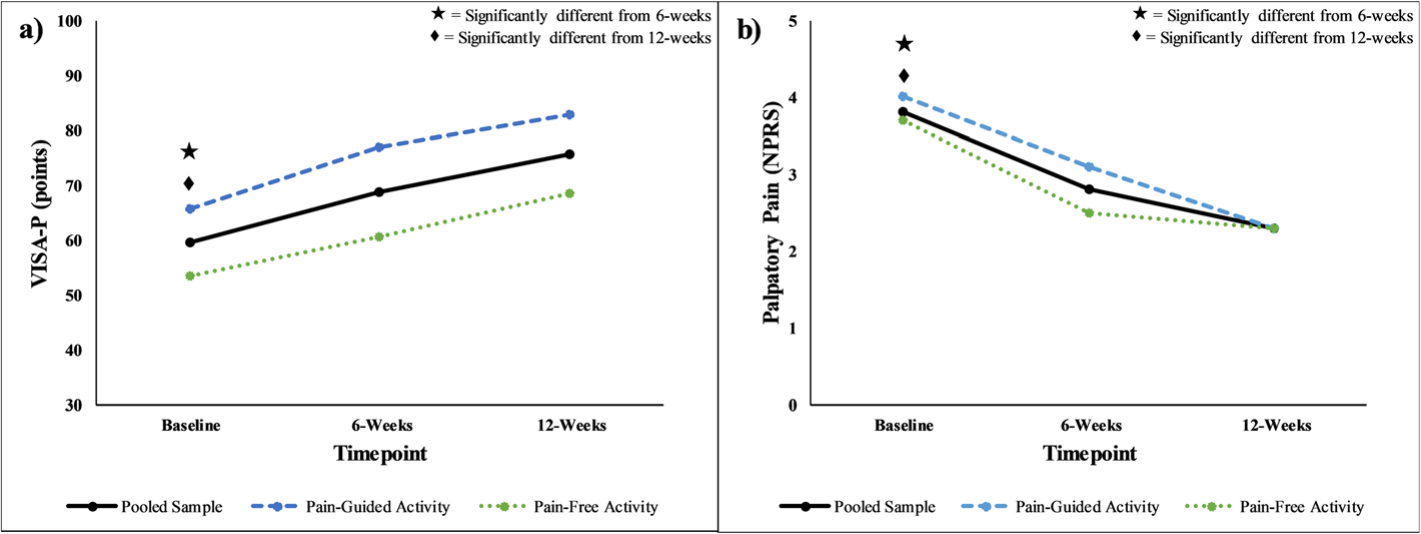
Estimated marginal means for **a** VISA-P and **b** palpatory pain for the pooled samples and groups. Note: Statistical significance is for the pooled sample only and Y-axis is truncated

There were significant effects of time and group for VISA-P, *p* = 0.005 and *p* = 0.029, respectively. There was a significant difference between baseline (*M* = 59.6, SE = 3.8) and both 6 week (*M* = 68.8, SE = 3.8) and 12 week (*M* = 75.6, SE = 4.0) follow-ups, *p* = 0.043 and *p* = 0.001, respectively (Fig. 4). There was no difference between 6- and 12-week follow-ups, *p* = 0.136. All differences between timepoints exceeded the SDC and differences between baseline and 12 weeks exceeded the MCID [22]. For the effect of group, scores for the PGA group (*M* = 75.1, SE = 3.6) was significantly higher than the PFA group (*M* = 60.8, SE = 4.5).

There was a significant effect of time, but not group, for palpatory pain, *p* = 0.007 and *p* = 0.769, respectively. There was a significant difference between baseline (*M* = 3.8, SE = 0.5) and both 6 week (*M* = 2.8, SE = 0.6) and 12 week (*M* = 2.3, SE = 0.6) follow-ups, *p* = 0.010 and *p* = 0.006, respectively (Fig. 4). All differences between timepoints exceeded the SDC but not the MCID [23, 52].

#### Quality of life

**Fig. 5:**
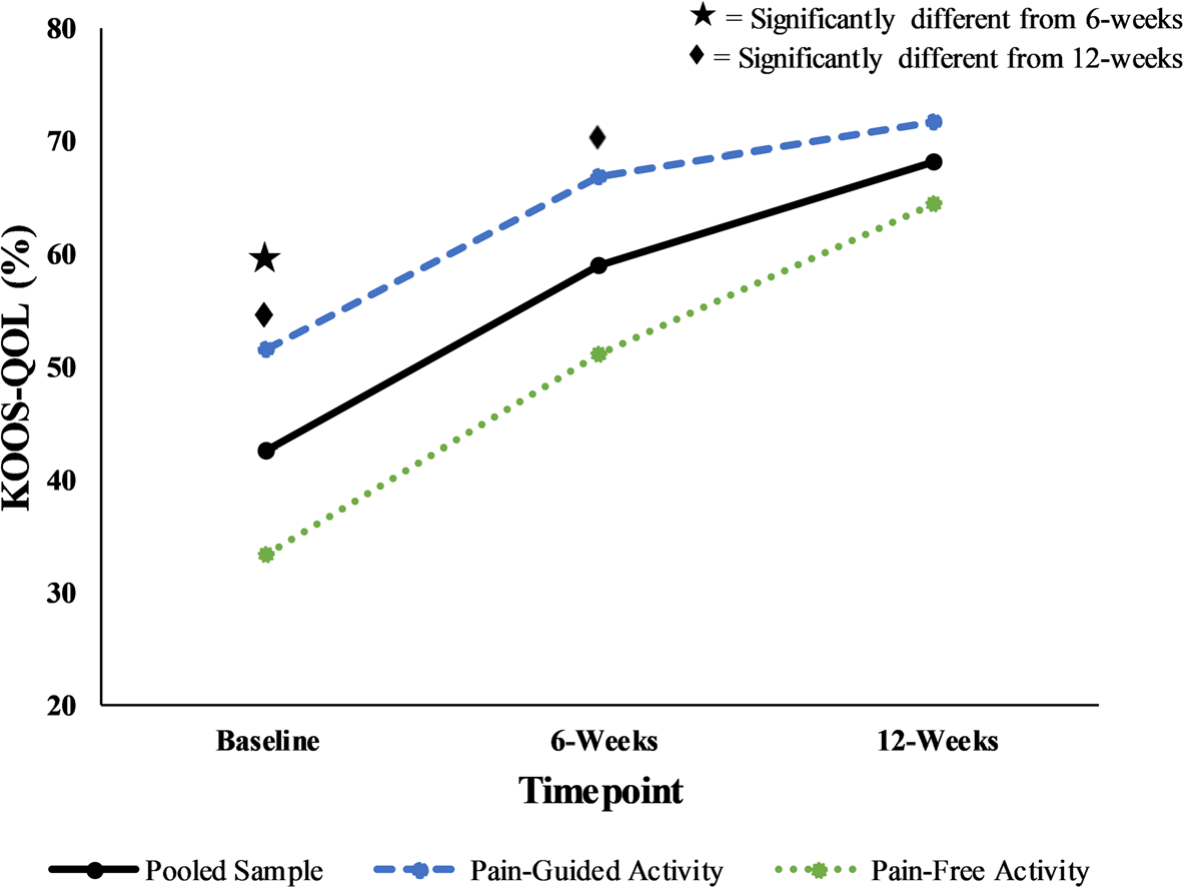
Estimated marginal means for KOOS-QOL for the pooled sample and groups. Note: Statistical significance is for the pooled sample only and Y-axis is truncated

There were significant effects of time and group for the KOOS-QOL, *p* < 0.001 and *p* = 0.041, respectively. There was a significant difference between baseline (*M* = 42.4, SE = 3.7) and both 6 week (*M* = 58.9, SE = 3.7) and 12 week (*M* = 68.0, SE = 3.9) follow-ups, *p* < 0.001 for both differences. Additionally, there was a significant difference between 6- and 12-week follow-ups (*p* = 0.029) (Fig. 5). All differences between timepoints exceeded the SDC [53]. For the effect of group, scores for the PGA group (*M* = 63.2, SE = 3.8) was significantly higher than PFA group (*M* = 49.6, SE = 4.7).

#### Psychological factors

**Fig. 6:**
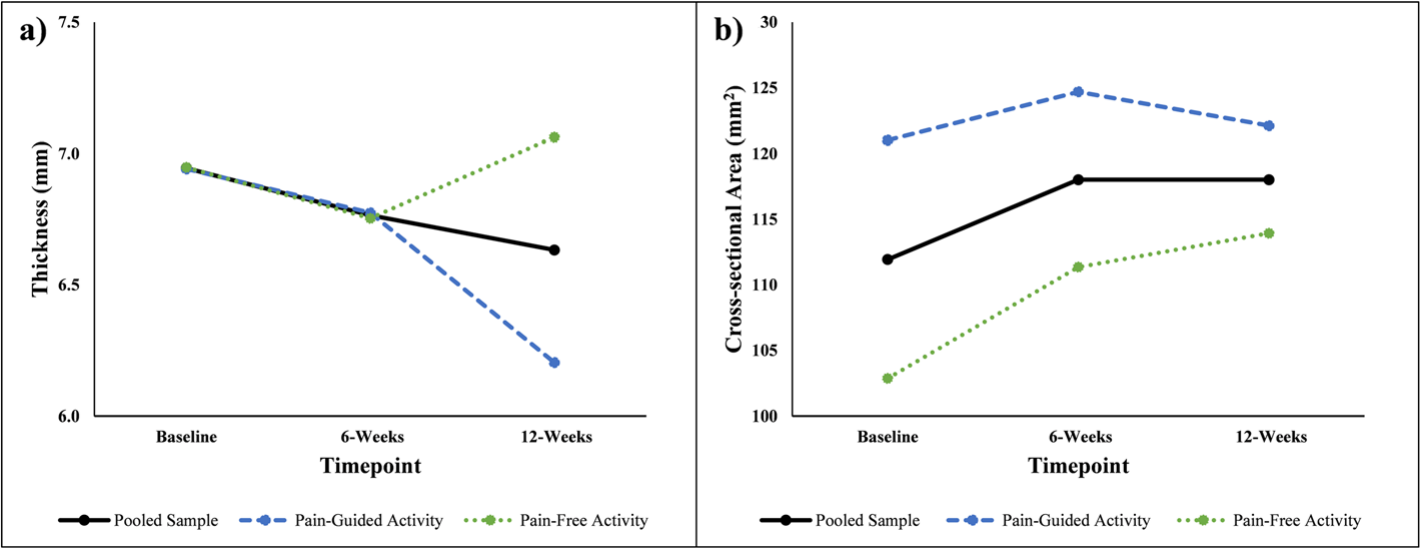
Estimated marginal means for the **a** TSK-17 and **b** DASS-21 for the pooled sample and groups. Note: Statistical significance is for the pooled sample only and the *Y*-axis is truncated

There was a significant effect of time, but not group, for the TSK, *p* = 0.045 and *p* = 0.25, respectively. There was a significant difference between baseline (*M* = 36.4, SE = 1.1) and 12 weeks (*M* = 34.2, SE = 1.1), *p* = 0.015 (Fig. 6). There was no difference between baseline and 6 weeks (*p* = 0.45) or 6 weeks and 12 weeks (*p* = 0.08). All differences between timepoints exceed the SDC.

For the PCS, the assumption of normality was not met after removal of outliers or log transformation of the outcome. Therefore, significance testing was not completed, and the median and interquartile range is reported in Table 3. PCS scores were similar across timepoints and between groups.

In the initial analysis, two data points were identified as outliers for the DASS-21. After removal the assumption of normality were met. There was a significant effect of group, but not time, for the DASS-21, *p* = 0.005 and *p* = 0.08, respectively (Fig. 6). The PGA group (*M* = 2.7, SE = 1.1) was significantly lower than the PFA group (*M* = 5.5, SE = 1.4). Although, differences between baseline and 12 weeks and 6 weeks and 12 weeks exceeded the SDC.

#### Tendon morphology

**Fig. 7:**
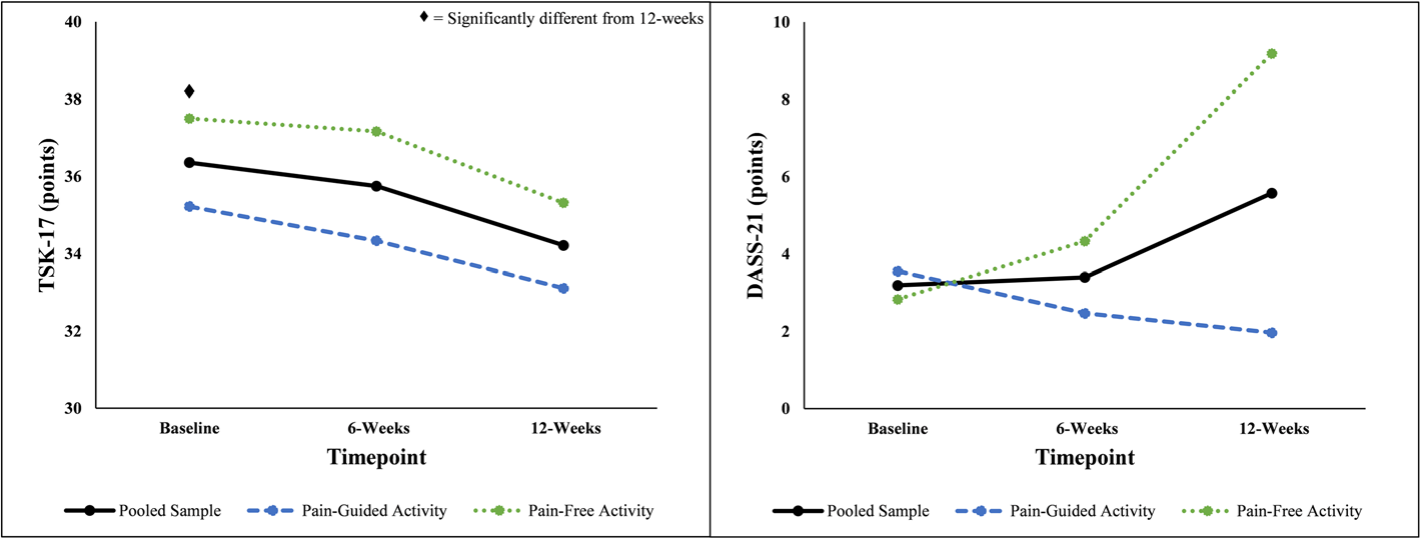
Estimated marginal means for **a** tendon thickness and **b** cross-sectional area for the pooled sample and groups. Note: *Y*-axis is truncated

In the initial analysis, two outliers were identified for CSA. After removal of these outliers, all assumptions of normality were met. The effects of time and group were not significant for thickness (time: *p* = 0.55; group: *p* = 0.79) or CSA (time: *p* = 0.57; group: *p* = 0.54) (Fig. 7). For thickness, differences from baseline to 6 weeks and 12 weeks exceeded the SDC. Differences between timepoints did not exceed the SDC for CSA.

#### Tendon mechanical properties

**Fig. 8:**
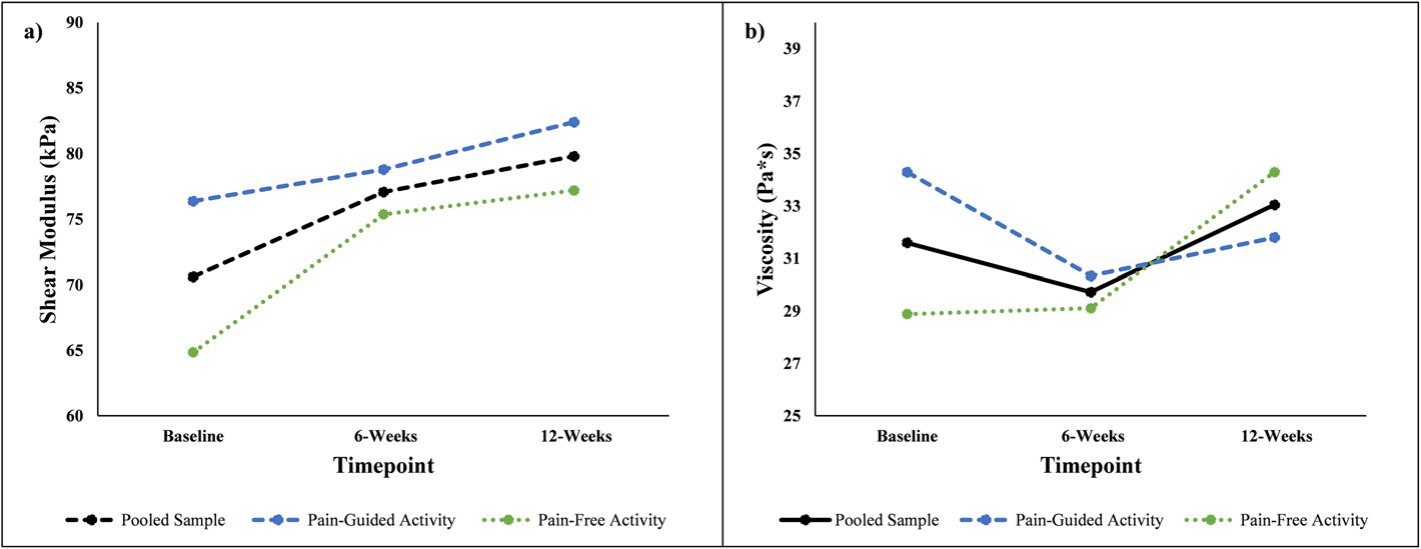
Estimated marginal means for **a** shear modulus and **b** viscosity for the pooled sample and groups. Note: *Y*-axis truncated

The effects of time and group were not significant for static shear modulus (time*: p* = 0.61; group: *p* = 0.50) or viscosity (time: *p* = 0.48; group: *p* = 0.75) (Fig. 8). Differences between timepoints did not exceed the SDC for shear modulus. All differences between timepoints exceeded the SDC for viscosity.

#### Lower extremity function

**Fig. 9:**
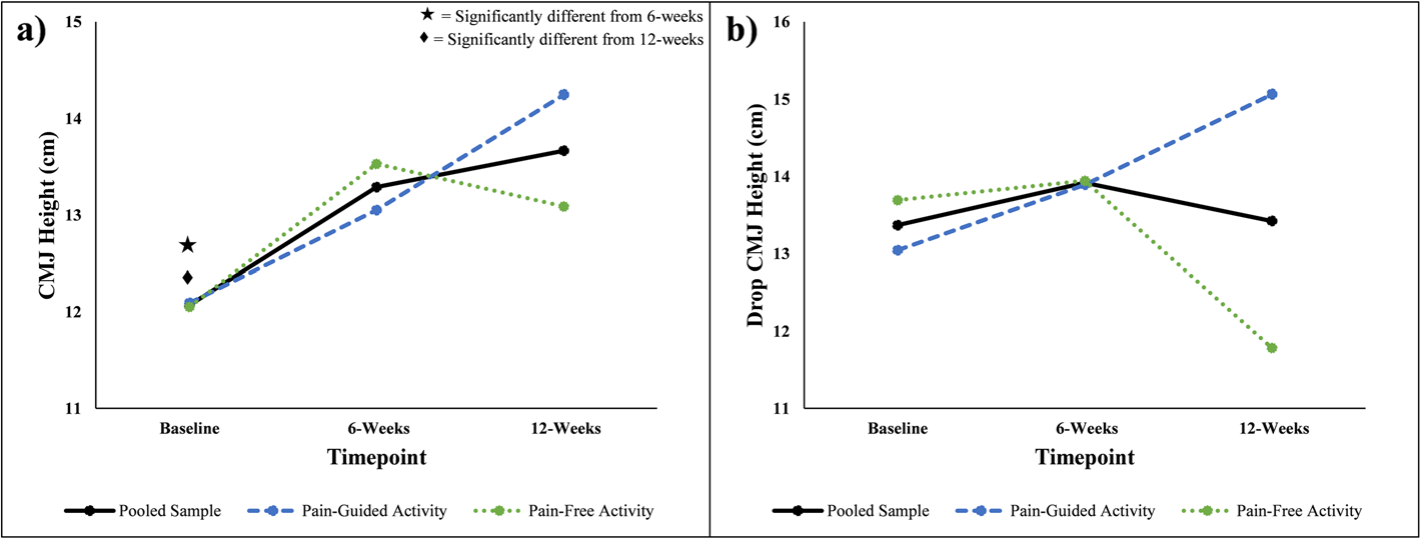
Estimated marginal means for **a** CMJ and **b** drop CMJ height for the pooled sample and groups. Note: Statistical significance is for the pooled sample only and the *Y*-axis is truncated

There was a significant effect of time, but not group, for CMJ height, *p* = 0.046, *p* = 0.92, respectively. There was a significant difference between baseline (*M* = 12.1, SE = 1.2) and both 6-week (*M* = 13.3, SE = 1.2) and 12-week (*M* = 13.7, SE = 1.3) follow-ups, *p* = 0.033 and *p* = 0.045, respectively (Fig. 9). There was no difference between 6-week and 12-week follow-ups, *p* = 0.62. Differences from baseline to 6-week and 12-week follow-ups exceeded the SDC.

The effects of time and group were not significant for drop CMJ height, *p* = 0.70 and *p* = 0.73, respectively (Fig. 9). Differences between timepoints did not exceed the SDC.

#### Quadriceps muscle performance

**Fig. 10:**
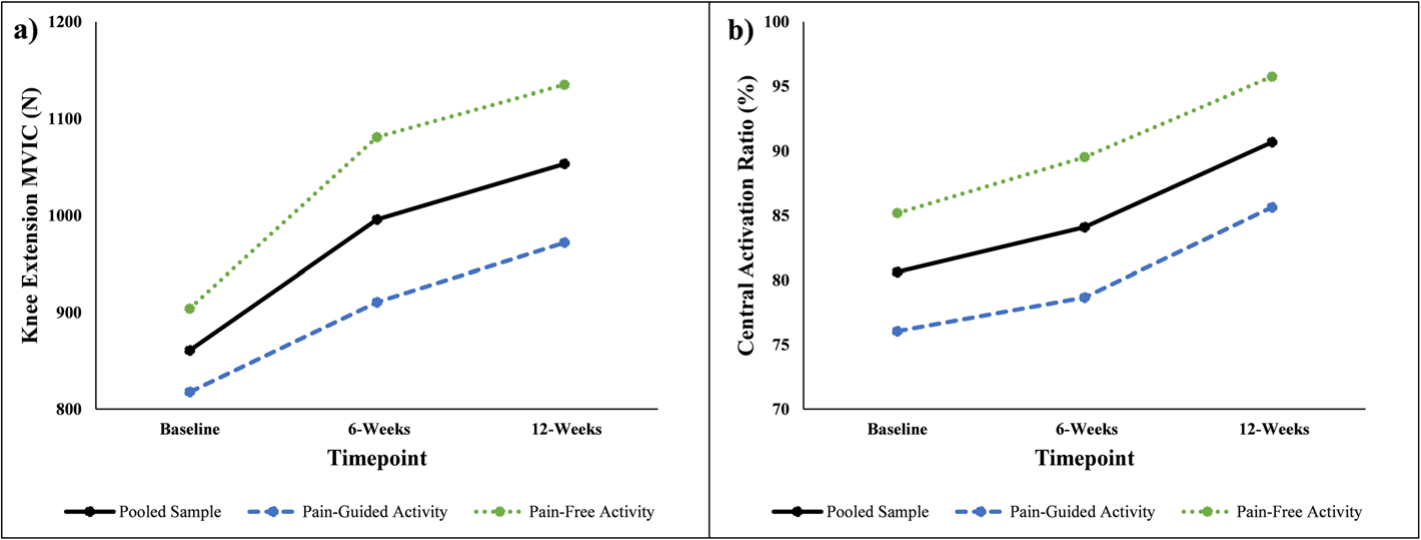
Estimated marginal means for **a** knee extension MVIC and **b** central activation ratio for the pooled sample and groups. Note: *Y*-axis is truncated

The effects of time and group were not significant for knee extension MVIC (time: *p* = 0.15; group*: p* = 0.08) or CAR (time: *p* = 0.20; group: *p* = 0.10) (Fig. 10). All differences between timepoints exceeded the SDC for both measures.

## Discussion

This pilot and feasibility randomized clinical trial had two aims. The first was to assess the feasibility of using the Pain-Monitoring Model to guide activity modification during treatment for patellar tendinopathy. The second was to evaluate whether the planned outcome measures would be able to detect differences in several areas of tendon health over the course of treatment. A total of 108 individuals were screened and 15 individuals were randomized. Retention was high, with only one missed evaluation session. Compliance with activity restrictions was excellent regardless of group, while compliance with treatment and training diaries was more variable. The PGA group had higher compliance with treatment and training diaries than the PFA group. In the compete sample, VISA-P scores, palpatory pain, KOOS-QOL scores, TSK scores, and CMJ height improved significantly and changes exceeding the SDC were detected for at least one outcome measure in each domain of tendon health.

### Recruitment and randomization

Fifteen of the 30 planned participants were consented and randomized to an intervention group in the ~ 13-month recruitment window. Although this fell short of our recruitment goal, the rate of recruitment was not consistent throughout the recruitment window. In the first 9 months of recruitment, six participants joined the study compared to nine participants in the final four months (Supplementary Figure S.1). This increase in recruitment toward the end of the study can be attributed to identification of new community collaborations, primarily adult recreational sports leagues with a large social media and community presence. In the full randomized clinical trial, we would use a non-inferiority design where the primary hypothesis is that the pain-monitoring group would not be substantially worse than the pain-free group at 12 weeks [54]. In other words, we would need to be adequately powered to detect a minimally clinically important difference of 13 points in the VISA-P score at 12 weeks. With alpha set at 0.025 and 80% power, we would need 32 participants per group and would recruit 80 participants to account for drop-out. At our recruitment rate in the final 4 months, it would take approximately 3 years to recruit all participants, which we deemed to be a realistic timeline.

The current randomization scheme resulted in uneven group assignment since we did not reach our target recruitment. Therefore, when conducting the full RCT, the randomization should include blocking to ensure relatively equal assignment throughout the study. Additionally, there was inadequate allocation concealment, as the randomization scheme was stored as a spreadsheet, which was accessible by the member of the research team that was recruiting and consenting participants. This has the potential for introducing bias, as this member of the research team may be aware of future assignments during recruitment. A full clinical trial should utilize computer based allocation concealment, as described by Vickers, so that members of the research team are not aware of assignment until after the participant has been consented [55]. Despite these limitations, the randomization scheme performed well, as the baseline characteristics were similar between groups.

It is also important to note that fourteen of the fifteen participants competed in sport at the recreational level and the remaining participant competed at the collegiate level. The lack of higher level athletes was surprising, given that the prevalence of patellar tendinopathy increases with competition level [56, 57]. Detailed reasons for declined participation are not available; however, we did note that several participants that chose not to participate expressed concern about missing organized team activities and competitions. Thus, it is likely that higher level athletes self-selected to not participate, which limits generalizability to populations other than recreational athletes. Prior to a full clinical trial, additional effort should be made in building relationships with coaches and sports medicine professionals to ensure that the study sample is more representative of the population with patellar tendinopathy.

### Compliance and retention

A priori compliance targets were not established. However, compliance outcomes can be compared to prior clinical trials using similar methods. The Heavy-Slow Resistance protocol was originally described and utilized in an RCT by Kongsgaard et al. [11]. In their study, the mean compliance rate for treatment was 91 ± 5%. Compliance rates were slightly lower for the PGA group (86.1 ± 13.0%) and substantially lower in the PFA group (67.1 ± 30.7%) and variability in compliance was larger for both groups than in the previous study. Additionally, Kongsgaard et al. had no drop-outs or losses to follow-up at their 12-week timepoint, while we had one participant lost to follow-up. In the prior study, they found that 70% of participants were satisfied with their clinical outcome at 12 weeks. Although the question wording was not identical, our participants mean satisfaction with treatment was 9.3 out of 10 points and 8.4 out of 10 points for the PGA and PFA groups, respectively. It’s important to note that compliance may have been influenced by the COVID-19 pandemic. For example, the participant lost to follow-up had been scheduled for their 12-week evaluation, but it had to be cancelled due to a hold on human subjects research. After this cancellation, we were unable to contact the individual to complete the online portion of the evaluation. Additionally, this individual did not provide their training diaries so only treatments supervised by a member of the research team could be counted when assessing treatment compliance. However, they had reported compliance with their home exercises at prior treatments, so their compliance was likely higher than recorded.

Training diaries are widely used in orthopedic research to assess compliance but the completion rate of these training diaries is rarely reported [8, 9, 15, 58–60]. Therefore, it is challenging to compare our compliance rates with training diaries and activity modification to prior studies. A recent pilot and feasibility study for plantar fasciopathy by Riel et al. followed compliance with a home exercise program for 8 weeks using training diaries [61]. Their reported completion rate was 75%. Our completion rate was substantially higher for the PGA group and slightly higher for the PFA group. To our knowledge, no prior studies have reported compliance with activity restrictions using the pain-monitoring model or with other forms of activity modification in patellar tendinopathy. Participants completing training diaries tend to overestimate their compliance with interventions but have higher compliance than if training diaries are not required [62]. Thus, the primary purpose of our training diaries was to encourage compliance, with awareness that the values may not reflect participant’s true compliance. Additionally, this is more in line with clinical practice, where clinicians provide recommendations to patients but cannot assess how closely the patients adhere to those recommendations. Other forms of activity tracking were explored to monitor compliance; however, activity trackers are not able to capture pain levels. Therefore, they would not be able to identify if participants followed activity restrictions.

### Safety

Assessing safety is another key component of pilot and feasibility trials. In our study, we had two adverse events. In both instances, participants experienced a new onset of lateral knee pain, which were aggravated by the treatment exercises. Given that the symptoms initially appeared while completing activities unrelated to study participation, it is unlikely that these events can be attributable to the study interventions.

### Clinical outcomes

The purpose of pilot and feasibility studies is not to assess the efficacy or effectiveness of an intervention, in this case, activity modification [16]. Thus, we intentionally did not test the group by time interaction to determine if the magnitude of change in outcome measures was different between groups. However, it is of interest to determine if the proposed outcome measures have adequate sensitivity to capture the effect of the interventions [16]. Therefore, we analyzed the effect of time in the pooled sample and compared mean differences between timepoints to the SDC and MCID of the respective measures.

Significant differences between timepoints were observed for VISA-P scores, palpatory pain, KOOS-QOL scores, TSK scores, and CMJ height, indicating that the Heavy-Slow Resistance protocol had an appreciable effect on symptom severity, knee-related quality of life, fear of movement, and lower extremity function. Therefore, this treatment protocol appears appropriate for use in a larger RCT. Furthermore, differences between at least two timepoints exceeded the SDC for all outcome measures, except for the PCS, CSA, static shear modulus, and drop CMJ height. Thus, we have at least one outcome measure for each domain of tendon health that can potentially detect the effect of our interventions. Finally, since the sample was unbalanced with a larger proportion of participants in the PGA group, the significant improvements in symptom severity and lower extremity function supports the plausibility that pain-guided activity may not be detrimental to recovery during exercise therapy. Therefore, a full RCT is warranted.

A potential benefit of utilizing a pain-guided approach to activity is that it may reduce the negative psychological consequences of injury [13]. However, scores on the DASS-21 were low, indicating low levels of depression, anxiety, and/or stress. Furthermore, when comparing individual sub-scores to established cut-offs, none of the participants had clinically meaningful levels of depression, anxiety, and/or stress at baseline [63, 64]. This may be due in part to the nature of the participants recruited. Only one participant was currently playing a sport at the collegiate level or above. Individuals that participate in sport at higher levels of competition typically derive more of their social identity and self-worth from their role as an athlete [65]. Thus, they may suffer a greater degree of negative psychological consequences in response to injury. This suggests that in a full RCT, greater effort should be made in recruiting high level athletes and stratification for competition level may be necessary during randomization.

Although depression, anxiety, and stress were not prevalent in this sample, kinesiophobia levels were high. At baseline, 7 out of 15 participants met or exceed a previously established threshold (37 points) for “high kinesiophobia” and all participants scored a 30 or above [66]. Additionally, TSK scores decreased from baseline to 6 and 12 weeks. These changes were significantly different from baseline to 12 weeks and all differences between timepoints exceeded the SDC. Therefore, it may be of interest to track this metric in full RCT to determine how activity modification recommendations impact fear of movement and re-injury.

### Limitations

This study has several limitations, including uneven randomization, lack of allocation concealment, and only one high-level athlete, which were discussed previously. Additionally, examiners were not blinded to group assignment, which may introduce bias when collecting baseline and follow-up measures. It is not desirable to blind the treating physical therapist to group assignment, as we believe that communication between the clinician and the participant about their weekly activity will promote adherence to activity modification. Since this was a pilot and feasibility study, one member of the research team performed all treatments and evaluations, so it was not possible to keep the examiner blinded. Another limitation was the use of paper-training diaries. The primary purpose of these diaries was to promote adherence, not assess compliance. However, there is evidence that suggests that study participants are more diligent and honest when completing electronic diaries [67]. Thus, we may have greater confidence in the accuracy of their self-reported compliance. Additionally, use of electronic diaries would allow us to send reminders and limit lost data. Finally, seven participants were impact by COVID-19, which either limited their ability to complete treatment and/or follow-up evaluations. As a result, those participants that were still participating in treatment may not have received the same magnitude of loading as those that completed treatment prior to the pandemic. Also, the estimated marginal means for 12-week follow-ups was based on a reduced sample size, which may over- or under-estimate the magnitude of change in the missing outcome measures.

## Conclusions

The use of a pain-guided activity modification during treatment for patellar tendinopathy appears feasible, based on the recruitment, compliance, and retention observed in this study. Additionally, the proposed outcome measures appear appropriate, as we observed changes that exceeded the SDC for each domain of tendon health. Although significant improvements were detected in symptom severity and lower extremity function in the pooled sample, a larger, more stringent RCT is needed to assess the impact of pain-guided activity modification on clinical outcomes during exercises therapy for patellar tendinopathy.

## Supplementary Information

**Additional file 1.**

## Data Availability

The datasets used and/or analyzed during the current study are available from the corresponding author on reasonable request.

## Abbreviations

CAR: Central activation ratio
CMJ: Counter-movement jump
CSA: Cross-sectional area
cSWE: Continuous shear wave elastography
DASS-21: Depression, anxiety, and stress scale
GLMM: Generalized linear mixed model
KOOS-QOL: Knee injury and osteoarthritis outcome score–quality of life subscale
M: Estimated marginal mean
MCID: Minimally clinically important difference
MVIC: Maximal voluntary isometric contraction
NPRS: Numeric pain rating scale
PCS: Pain catastrophizing scale
PFA: Pain-free activity
PGA: Pain-guided activity
RCT: Randomized clinical trial
REDCap: Research electronic data capture
RM: Repetition max
SDC: Smallest detectable change
SE: Standard error
TSK-17: Tampa scale of kinesiophobia
VISA-P: Victorian institute of sports assessment–patellar tendon
