# Supplementary material for: Pain-guided activity modification during treatment for patellar tendinopathy: a feasibility and pilot randomized clinical trial
Source: Pilot Feasibility Stud. 2021 Feb 25;7:58. doi: 10.1186/s40814-021-00792-5 (PMC7905015; doi:10.1186/s40814-021-00792-5)
Supplement: Supplementary file 1 — Additional file 1. [file 40814_2021_792_MOESM1_ESM.docx]

**Supplementary Material**

Recruitment Strategy For Pilot and Feasibility Study

The recruitment strategy was developed to reach a wide audience through a variety of media formats to ensure a representative sample of participants with patellar tendinopathy. Beginning in January, 2019, local orthopedic and sports medicine physicians, as well as the University of Delaware Physical Therapy clinic, were notified of study initiation and provided with consent to contact forms and study flyers. The flyers directed interested individuals to an online REDCap survey where they could submit contact information and answer preliminary screening questions. On February 26^th^, 2019, additional flyers were posted in athletic centers and gyms throughout New Castle County, DE. Facebook and Instagram advertisements were begun on May 9^th^, 2019, with a target audience of sports medicine professionals, coaches, and adults within the target age range with interests in sports or recreational fitness. On September 1^st^, 2019, advertisements were initiated with Delaware Sports League, an adult recreational sports league. These advertisements included monthly emails and dedicated social media posts. Delaware Sports League advertisements ran from September through November. The total number of interested individuals and the outcomes of screening are displayed in Figure S.1.


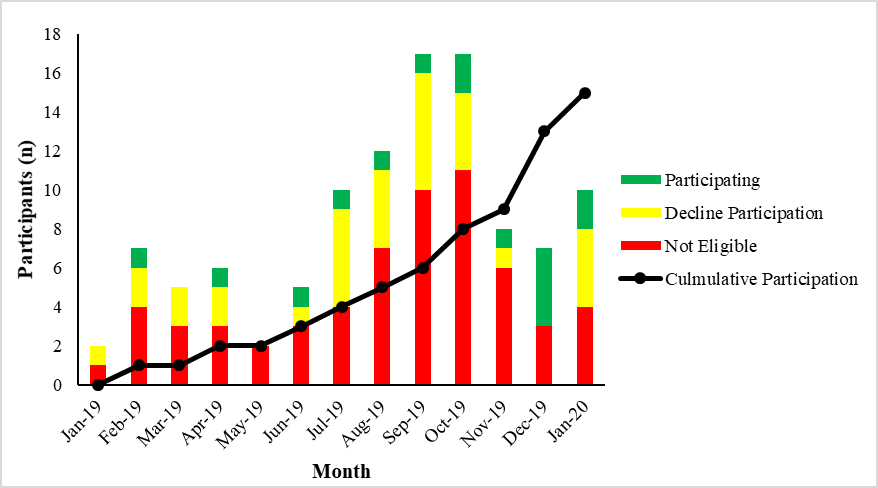


**Figure S.1.** Recruitment by month for pilot and feasibility randomized clinical trial.

**Table S.1.** Reliability and responsiveness of outcome measures.

|  | **ICC** | **SEM** | **SDC_95%-individual_** | **SDC_95%-group_** | **MCID** |
| --- | --- | --- | --- | --- | --- |
| **VISA-P (points)**(1) | 0.95 | 4.0 | 11.1 | 1.2 | 13.0 |
| **NPRS (points)**(2,3) | 0.95 | 0.48 | 1.3 | 0.12 | 1.7 |
| **KOOS-QOL (%)**(4) | 0.95 | 2.6 | 7 | 1.1 | N.E. |
| **TSK-17 (points)**(5,6) | 0.91 | 3.3 | 9.2 | 0.7 | N.E. |
| **PCS (points)**(7) | N.E | N.E | 15.2 | 1.0 | 5.8 |
| **DASS-21 (points)**(8) | N.E. | N.E. | 10.12 | 0.26 | N.E. |
| **Tendon Thickness (mm)**(9) | 0.82 - 0.96 | 0.21 | 0.59 | 0.16 | N.E. |
| **Tendon CSA (mm^2^)** | 0.878 – 0.882 | 11.2 | 31.0 | 11.7 | N.E. |
| **Static Shear Modulus (kPa)** | 0.664 – 0.742 | 14.1 – 21.1 | 39.1 – 58.6 | 10.8 – 16.3 | N.E. |
| **Viscosity (Pa*sec)** | 0.874 – 0.877 | 1.4 – 4.9 | 3.8 – 13.6 | 1.1 – 3.8 | N.E. |
| **CMJ Height (cm)**(10) | 0.91 | 1.44 | 3.99 | 0.73 | N.E. |
| **Drop CMJ Height (cm)** (10) | 0.88 – 0.92 | 1.66 | 4.61 | 0.84 | N.E. |
| **Knee Extension MVIC (% change)**(11) | 0.98 | 5.67 | 15.72 | 2.87 | N.E. |
| **Quadriceps CAR (%)**(12) | 0.97 | 2.36 | 6.55 | 2.07 | N.E. |

ICC = Intraclass correlation coefficient, SEM = Standard error of measure, SDC = Smallest detectable change, MCID = Minimally clinically important difference, VISA-P = Victorian Institute of Sport Assessment – Patellar Tendon questionnaire, NPRS = Numeric Pain Rating Scale, KOOS-QOL = Knee injury and Osteoarthritis Outcome Score – Quality of Life subscale, TSK = Tampa Scale of Kinesiophobia, PCS = Pain Catastrophizing Scale, CSA = Cross-sectional area, CMJ = Counter-movement jump, MVIC = Maximum voluntary isometric contraction, CAR = Central activation ratio, N.E. = Not established. Note: Reliability and responsiveness values for tendon CSA, shear modulus, and viscosity are from unpublished studies, which are currently in submission, conducted by the authors (ALS, KGS, CC, and RTP).

**Table S.2**. Effects of group and time of generalized linear mixed model for outcome measures in Aim 4 prior to outlier removal.

| **Category** | **Outcome** | **Time** | | **Group** | |
| --- | --- | --- | --- | --- | --- |
|  |  | **F** | **p** | **F** | **p** |
| **Symptoms** | VISA-P | 6.587 | **0.005** | 6.064 | **0.029** |
|  | Palpatory Pain | 6.508 | **0.007** | 0.90 | 0.769 |
| **Quality of Life** | KOOS – QOL | 21.976 | **<0.001** | 5.136 | **0.041** |
| **Psychological Factors** | TSK | 3.528 | **0.045** | 1.428 | 0.253 |
|  | DASS-21 | 2.806 | 0.079 | 0.581 | 0.459 |
| **Morphology** | Thickness | 0.618 | 0.549 | 0.074 | 0.790 |
|  | CSA | 0.229 | 0.797 | 0.220 | 0.647 |
| **Mechanical Properties** | Shear Modulus | 0.512 | 0.606 | 0.487 | 0.496 |
|  | Viscosity | 0.771 | 0.476 | 0.109 | 0.746 |
| **Lower Extremity Function** | CMJ Height | 3.608 | **0.046** | 0.011 | 0.918 |
|  | Drop CMJ Height | 0.370 | 0.695 | 0.125 | 0.729 |
| **Quadriceps Muscle Performance** | MVIC | 2.068 | 0.150 | 3.743 | 0.078 |
|  | CAR | 1.787 | 0.199 | 3.326 | 0.099 |

CSA = cross-sectional area; CMJ = counter-movement jump; MVIC = maximal voluntary isometric contraction; CAR = central activation ratio

**Table S.3.** Effects of group and time of generalized linear mixed model for outcome measures in Aim 4 after outlier removal.

| **Category** | **Outcome** | **Time** | | **Group** | |
| --- | --- | --- | --- | --- | --- |
|  |  | **F** | **p** | **F** | **p** |
| **Symptoms** | VISA-P | 6.587 | **0.005** | 6.064 | **0.029** |
|  | Palpatory Pain | 6.508 | **0.007** | 0.90 | 0.769 |
| **Quality of Life** | KOOS – QOL | 21.976 | **<0.001** | 5.136 | **0.041** |
| **Psychological Factors** | TSK | 3.528 | **0.045** | 1.428 | 0.253 |
|  | DASS-21 | 2.800 | 0.081 | 6.678 | **0.005** |
| **Morphology** | Thickness | 0.618 | 0.549 | 0.074 | 0.790 |
|  | CSA | 0.579 | 0.570 | 0.403 | 0.536 |
| **Mechanical Properties** | Shear Modulus | 0.512 | 0.606 | 0.487 | 0.496 |
|  | Viscosity | 0.771 | 0.476 | 0.109 | 0.746 |
| **Lower Extremity Function** | CMJ Height | 3.608 | **0.046** | 0.011 | 0.918 |
|  | Drop CMJ Height | 0.370 | 0.695 | 0.125 | 0.729 |
| **Quadriceps Muscle Performance** | MVIC | 2.068 | 0.150 | 3.743 | 0.078 |
|  | CAR | 1.787 | 0.199 | 3.326 | 0.099 |

CSA = cross-sectional area; CMJ = counter-movement jump; MVIC = maximal voluntary isometric contraction; CAR = central activation ratio.

**Table S.3.** Estimated marginal means, standard error and pair-wise comparisons of timepoints for the pooled sample prior to outlier removal.

| **Category** | **Outcome** | **Baseline** | | **6-Weeks** | | **12-Weeks** | | **p-values** | | |
| --- | --- | --- | --- | --- | --- | --- | --- | --- | --- | --- |
|  |  | **M** | **SE** | **M** | **SE** | **M** | **SE** | **Baseline to 6-Weeks** | **Baseline to 12-Weeks** | **6-Weeks to 12-Weeks** |
| **Symptoms** | VISA-P (points) | 59.6 | 3.8 | 68.8 | 3.8 | 75.6 | 4.0 | **0.043** | **0.001** | 0.136 |
|  | Palpatory Pain (NPRS) | 3.8 | 0.5 | 2.8 | 0.6 | 2.3 | 0.6 | **0.010** | **0.006** | 0.347 |
| **Quality of Life** | KOOS-QOL (%) | 42.4 | 3.7 | 58.9 | 3.7 | 68.0 | 3.9 | **<0.001** | **<0.001** | **0.029** |
| **Psychological Factors** | TSK (points) | 36.4 | 1.1 | 35.8 | 1.1 | 34.2 | 1.1 | 0.448 | **0.015** | 0.075 |
|  | DASS-21 (points) | 3.2 | 1.4 | 4.3 | 1.4 | 6.2 | 1.4 | 0.383 | 0.027 | 0.146 |
|  | PCS (points)^#^ | 2.0 | 6.0 | 1.0 | 2.0 | 1.0 | 3.0 | - | - | - |
| **Morphology** | Thickness (mm) | 6.9 | 0.5 | 6.8 | 0.5 | 6.6 | 0.6 | 0.427 | 0.329 | 0.678 |
|  | CSA (mm^2^) | 115.3 | 10.8 | 118.0 | 10.8 | 119.7 | 12.0 | 0.613 | 0.558 | 0.821 |
| **Mechanical** | Shear Modulus (kPa) | 70.6 | 6.3 | 77.1 | 6.2 | 79.8 | 9.2 | 0.427 | 0.391 | 0.796 |
|  | Viscosity (Pa*sec) | 31.6 | 2.4 | 29.7 | 2.3 | 33.0 | 3.1 | 0.396 | 0.628 | 0.268 |
| **Lower Extremity Function** | CMJ Height (cm) | 12.1 | 1.2 | 13.3 | 1.2 | 13.7 | 1.3 | **0.033** | **0.045** | 0.620 |
|  | Drop CMJ Height (cm) | 13.4 | 1.2 | 13.9 | 1.3 | 13.4 | 1.4 | 0.423 | 0.953 | 0.588 |
| **Quadriceps Muscle Performance** | MVIC (N) | 860.9 | 56.7 | 995.9 | 60.0 | 1053.4 | 85.6 | 0.138 | 0.082 | 0.601 |
|  | CAR (%) | 80.6 | 3.3 | 84.1 | 3.8 | 90.7 | 4.9 | 0.440 | 0.076 | 0.250 |

CSA = cross-sectional area; CMJ = counter-movement jump; MVIC = maximal voluntary isometric contraction; CAR = central activation ratio. NPRS = numeric pain rating scale. ^#^PCS did not meet assumption of linearity so model significance was not tested and values reported are median and interquartile range, respectively. *n = 14 for patient reported outcome measures at 12-weeks (VISA-P, TSK, PCS, DASS-21) due to participant drop-out. n = 8 for all other measures at 12-weeks due to inability to complete in-person follow-ups because of COVID-19. **Bold** indicates statistically significant differences between timepoints.

**References:**

1. Hernandez-Sanchez S, Hidalgo MD, Gomez a. Responsiveness of the VISA-P scale for patellar tendinopathy in athletes. Br J Sports Med. 2012 Mar;3(6):453–7.

2. Alghadir AH, Anwer S, Iqbal A, Iqbal ZA. Test-retest reliability, validity, and minimum detectable change of visual analog, numerical rating, and verbal rating scales for measurement of osteoarthritic knee pain. J Pain Res [Internet]. 2018;11:851–6. Available from: http://www.ncbi.nlm.nih.gov/pubmed/29731662

3. Farrar JT, Young JP, LaMoreaux L, Werth JL, Poole RM. Clinical importance of changes in chronic pain intensity measured on an 11-point numerical pain rating scale. Pain [Internet]. 2001 Nov;94(2):149–58. Available from: http://www.ncbi.nlm.nih.gov/pubmed/11690728

4. Bekkers JEJ, de Windt TS, Raijmakers NJH, Dhert WJA, Saris DBF. Validation of the Knee Injury and Osteoarthritis Outcome Score (KOOS) for the treatment of focal cartilage lesions. Osteoarthr Cartil [Internet]. 2009 Nov;17(11):1434–9. Available from: http://dx.doi.org/10.1016/j.joca.2009.04.019

5. Lundberg M. Kinesiophobia - Various Aspects of Moving with Musculoskeletal Pain. Göteborg University; 2006.

6. Ostelo RWJG, Swinkels-Meewisse IJCM, Knol DL, Vlaeyen JWS, de Vet HCW. Assessing pain and pain-related fear in acute low back pain: what is the smallest detectable change? Int J Behav Med [Internet]. 2007;14(4):242–8. Available from: http://www.ncbi.nlm.nih.gov/pubmed/18001240

7. Pulles ANTD, Köke AJA, Strackke RP, Smeets RJEM. The responsiveness and interpretability of psychosocial patient-reported outcome measures in chronic musculoskeletal pain rehabilitation. Eur J Pain (United Kingdom). 2020;24(1):134–44.

8. Ruwaard J, Lange A, Schrieken B, Dolan C V., Emmelkamp P. The effectiveness of online cognitive behavioral treatment in routine clinical practice. PLoS One [Internet]. 2012;7(7):e40089. Available from: http://www.ncbi.nlm.nih.gov/pubmed/22792217

9. Del Baño-Aledo ME, Martínez-Payá JJ, Ríos-Díaz J, Mejías-Suárez S, Serrano-Carmona S, de Groot-Ferrando A. Ultrasound measures of tendon thickness: Intra-rater, Inter-rater and Inter-machine reliability. Muscles Ligaments Tendons J [Internet]. 2015;7(1):192–9. Available from: http://www.ncbi.nlm.nih.gov/pubmed/28717629

10. Silbernagel KG, Gustavsson A, Thomeé R, Karlsson J. Evaluation of lower leg function in patients with Achilles tendinopathy. Knee Surgery, Sport Traumatol Arthrosc [Internet]. 2006 Nov 3;14(11):1207–17. Available from: http://link.springer.com/10.1007/s00167-006-0150-6

11. Mentiplay BF, Perraton LG, Bower KJ, Adair B, Pua YH, Williams GP, et al. Assessment of lower limb muscle strength and power using hand-held and fixed dynamometry: A reliability and validity study. PLoS One. 2015;10(10):1–18.

12. Norte GE, Frye JL, Hart JM. Reliability of the superimposed-burst technique in patients with patellofemoral pain: A technical report. J Athl Train. 2015;50(11):1207–11.
